# Silver Nanoparticles: A Versatile Tool Against Infectious and Non-Infectious Diseases

**DOI:** 10.3390/antibiotics14030289

**Published:** 2025-03-11

**Authors:** Sara González-Fernández, Noelia Blanco-Agudín, David Rodríguez, Iván Fernández-Vega, Jesús Merayo-Lloves, Luis M. Quirós

**Affiliations:** 1 Department of Functional Biology, University of Oviedo, 33006 Oviedo, Spain; s.gonzalez@cinn.es (S.G.-F.); blancoanoelia@uniovi.es (N.B.-A.); 2 Instituto Universitario Fernández-Vega, Fundación de Investigación Oftalmológica, University of Oviedo, 33012 Oviedo, Spain; fernandezvivan@uniovi.es (I.F.-V.); merayojesus@uniovi.es (J.M.-L.); 3 Nanomaterials and Nanotechnology Research Center (CINN), Consejo Superior de Investigaciones Científicas, 33940 El Entrego, Spain; 4 Instituto de Investigación Sanitaria del Principado de Asturias (ISPA), 33011 Oviedo, Spain; 5 Department of Biochemistry and Molecular Biology, University of Oviedo, 33006 Oviedo, Spain; rodriguezdavid@uniovi.es; 6 Department of Pathology, Hospital Universitario Central de Asturias, 33011 Oviedo, Spain; 7 Department of Surgery, University of Oviedo, 33006 Oviedo, Spain

**Keywords:** silver nanoparticles, silver ions, antimicrobial, biofilm, diabetes, cancer, wound healing, biosensors

## Abstract

Silver nanoparticles possess remarkable properties that render them highly beneficial for medical applications in both infectious and non-infectious diseases. Among their most renowned attributes is their antimicrobial activity. They have demonstrated efficacy against a wide range of bacteria, fungi, protozoa, and viruses. Additionally, the antitumor and anti-diabetic properties of silver nanoparticles, along with their ability to promote wound healing and their application as biosensors, underscore their therapeutic potential for various non-infectious conditions. As silver nanoparticles are employed for medical purposes, their potential toxicity must be considered. While silver nanoparticles present a promising alternative in the therapeutic domain, further research is needed to elucidate their precise mechanisms of action, optimize their efficacy, and mitigate any potential health risks associated with their use.

## 1. Introduction

Silver nanoparticles (AgNPs) are nanomaterials with a size smaller than 100 nm [1]. Their large surface-area-to-volume ratio imparts unique electrical, optical, and catalytic properties, making them suitable for various applications, including the energy sector, the food industry, environmental protection, and biomedical therapies [2]. Specifically, AgNPs are known for their potent antimicrobial effects against bacteria, fungi, protozoa, and viruses [3,4,5,6].

**Figure 1:**
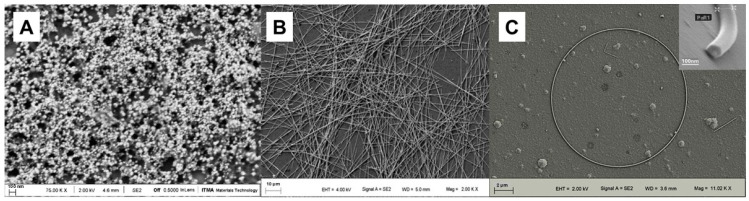
Electron micrographs of different types of AgNPs, showing significant differences in size and morphology. (**A**) Nanospheres (avg. dia. = 80 nm); (**B**) nanowires (avg. dia. = 100 nm, avg. lgth. = 100 µm); (**C**) nanorings (avg. dia. = 14 µm). Images (**A**,**C**) taken from González-Fernández et al. [1].

The biocidal effect of AgNPs is influenced by their size, morphology, surface properties, and concentration [7]. Generally, smaller AgNPs tend to exhibit greater efficacy, and in numerous studies, nanoparticles of smaller sizes have displayed the most significant antibacterial effects [8,9]. However, the relationship between size and efficiency is complex, and morphology constitutes another critical factor, though effectiveness depends on the interaction quality between the bacterial membrane and the nanoparticles. For instance, nanocubes exhibit stronger antibacterial effects compared to nanospheres and nanowires, as transmission electron microscopy reveals closer interaction between bacteria and nanocubes, resulting in enhanced antibacterial properties [7]. Similarly, studies on the antimicrobial effect of silver nanorings, nanowires, and nanospheres over prolonged periods have shown that in some cases, nanorings exhibited superior activity [1]. These data demonstrate how, in addition to size, particle morphology is critical for biological activity (Figure 1).

The presence of stabilizers on the surface of AgNPs alters their biological activity. The chemical structure of these agents allows for the manipulation of the electrokinetic characteristics of AgNPs, and biologically active compounds improve AgNP penetration through cell membranes [8].

To better understand and categorize these diverse materials, we propose a classification system based on the key physicochemical properties influencing their biocidal efficacy (Table 1).

The overuse of antibiotics has led to the emergence of resistance against nearly all available antibiotics. In 2019, over 1.2 million deaths were attributed to infections caused by multi-antibiotic-resistant bacteria. Consequently, the World Health Organization has recognized antibiotic resistance as a critical health issue. Given the difficulty in developing new antibiotics, alternative strategies are essential [10]. AgNPs have been investigated as a promising approach to prevent infections, decontaminate medical equipment, and treat ongoing infections [11].

In nature, bacteria rarely exist in their planktonic form; they typically form multicellular structures called biofilms, which offer advantages such as increased antibiotic resistance and stronger surface adhesion. Biofilm-associated infections are challenging to eradicate, as some persistent bacteria survive treatment. AgNPs have been shown to successfully inhibit biofilm formation over extended periods [12].

Beyond infections, AgNPs are useful in combating other conditions, such as cancer, diabetes, and inflammatory processes like wound healing [13,14,15]. This review will provide more information on these topics.

The increasing use of AgNPs for therapeutic purposes in various diseases underscores the need to study their potential cytotoxic impacts on humans. Therefore, it is crucial to thoroughly analyze the mechanisms by which AgNPs exert their biocidal activity, and how they are accumulated and eliminated from the body [11].

## 2. AgNP Synthesis

AgNPs are synthesized though a wide range of methodologies. The principal strategies are physical, chemical, and biological methods. The chemical synthesis of AgNPs represents a highly convenient, efficient, and straightforward approach for controlled production. Among these methods, chemical reduction is the most widely used technique, using both organic and inorganic reducing agents to achieve AgNP formation. This process typically involves the reduction of silver ions (Ag^+^) in aqueous or non-aqueous media, through the action of agents such as sodium borohydride, sodium citrate, ascorbate, polyol derivatives, and molecular hydrogen, among others. During this synthesis, these agents facilitate the reduction of Ag^+^ to elemental metallic silver (Ag^0^), allowing precise control over nanoparticle size, morphology, and stability [16]. However, these chemical agents raise concerns due to their toxicity and potential to cause environmental damage [17].

The photochemical method involves irradiating a solution containing metal precursors with ultraviolet or visible light to induce AgNP formation. The photochemical reduction method offers various advantages over conventional synthesis techniques, as it eliminates the requirement for toxic reagents, while circumventing the need for specialized instrumentation or advanced technical expertise. Furthermore, the process can be conducted under ambient conditions (atmospheric pressure and room temperature), which simplifies experimental protocols and facilitates energy-efficient synthesis. These conditions make photochemical synthesis an environmentally friendly and economically viable strategy for producing AgNPs with controlled properties [18].

Physical techniques are employed to produce AgNPs without the introduction of chemical agents. Evaporation–condensation and laser ablation can be considered the predominant physical methods for AgNP synthesis. These techniques offer outstanding advantages over chemical synthesis routes, notably, the elimination of solvent-derived impurities in fabricated thin films and the achievement of homogeneous AgNP dispersion [19]. However, these approaches are characterized by huge energy requirements, and may not be suitable for large-scale production [17].

Biological synthesis, or biosynthesis, employs microorganisms, plant extracts, or enzymes to reduce Ag^+^ in AgNPs. This method is environmentally friendly and sustainable, as it avoids toxic chemicals and harsh conditions. For instance, *Lacticaseibacillus rhamnosus* is able to generate stable and biocompatible AgNPs that also present antibacterial and photocatalytic properties [20].

Bacteria can produce several enzymes in broth culture, including reductases, which are able to accomplish AgNP biosynthesis. During the process, reductases act as reducing entities, transforming Ag^+^ to Ag^0^. This is a convenient solution, due to their quick replication and simple cultivation [21].

## 3. AgNPs in Infectious Pathologies

### 3.1. Antimicrobial Effects

In order to facilitate the reader’s understanding, a table with the therapeutic properties and mechanisms of action of AgNPs related to infectious pathologies, along with references, is included below (Table 2).

#### 3.1.1. Effects of AgNPs Against Bacteria

Although this process has been studied for years, the exact mechanism by which AgNPs impact bacterial viability is not yet fully understood, perhaps due to the numerous processes occurring simultaneously when bacteria interact with AgNPs [22]. It is accepted that the mechanism of action of AgNPs relies on the effect of the nanoparticles themselves and the release of Ag^+^.

Silver is positively charged, and therefore, Ag^+^ tends to interact with negatively charged biomolecules that are prevalent in cells, such as sulfur and phosphorus present in proteins, DNA, and cell membranes. This interaction causes damage to the cell wall and various morphological alterations in both Gram-positive and Gram-negative bacteria [24]. Moreover, the continuous release of Ag^+^ allows AgNPs to exert their action over extended periods [7].

It appears that Gram-negative bacteria are more sensitive than Gram-positive ones. Gram-positive bacteria have a thicker cell membrane and negatively charged teichoic acids, which can act as a barrier to AgNP penetration [7].

The size of AgNPs is crucial to their antibacterial efficacy, as a reduction in their dimensions enhances their ability to infiltrate bacterial structures [24]. AgNPs may penetrate the outer bacterial membrane and accumulate in the inner membrane or plasma membrane. Interestingly, it has been demonstrated that the main damage to the Gram-negative wall occurs at the plasma membrane, rather than the bacterial outer membrane [25]. The accumulation of nanoelements and Ag^+^ destabilizes the cell, ultimately enhancing membrane permeability, leading to the release of cellular contents, and, in the end, cell death. They can also interact with the lipid bilayer, causing fluidization of the membrane and altering its hydrophobicity. Consequently, membrane pores emerge, allowing AgNPs and Ag^+^ to penetrate the cell interior. This process causes certain cytosolic components to exit the cell through these newly created pores [22]. Furthermore, AgNPs interact with proteins from the cell membrane, such as efflux pumps, generating structural damage and subsequently causing membrane rupture [11].

When AgNPs enter the cell, they disrupt cell structures and cause the inactivation of respiratory enzymes. These events lead to the accumulation of reactive oxygen species (ROS) and the inhibition of ATP production. Protein synthesis is also altered, as AgNPs may denature ribosomes and disrupt bacterial signal transduction. AgNPs can remove phosphate groups from tyrosine residues located on peptide surfaces. Concurrently, Ag^+^ produces ROS, which eventually trigger the creation of superoxide radicals and hydrogen peroxide, among other compounds [22,23,24,25]. Hydroxyl radicals have the most deleterious impact on cell viability.

The reduction of O_2_ causes the generation of O_2_^−^, which can be considered a precursor of ROS. Enzymes known as superoxidedismutases facilitate the biochemical reaction O_2_^−^ + O_2_^−^ → H_2_O_2_ + O_2_, significantly increasing the rate constant associated with this reaction by several orders of magnitude. Genetic deletion of superoxidedismutases has led to lethal mutations, thereby emphasizing the indispensable role of this enzyme family [26]. H_2_O_2_ can be completely reduced, producing H_2_O, or partially reduced, producing OH. OH contributes to the oxidation reaction cascade. Alternatively, O_2_^−^ can engage in a reaction with nitric oxide radicals (NO) to produce reactive nitrogen species, including peroxynitrite anions (ONOO^−^), nitrogen oxide radicals (NO_2_^−^), nitrate anions (NO_3_^−^), and carbonate anions (CO_3_^−^). This oxidative mechanism has the potential to eliminate microorganisms, provided that the accumulation of hydroxyl radicals is effectively regulated, and it can result in oxidative damage to proteins, lipids, and nucleic acids [27].

**Figure 2:**
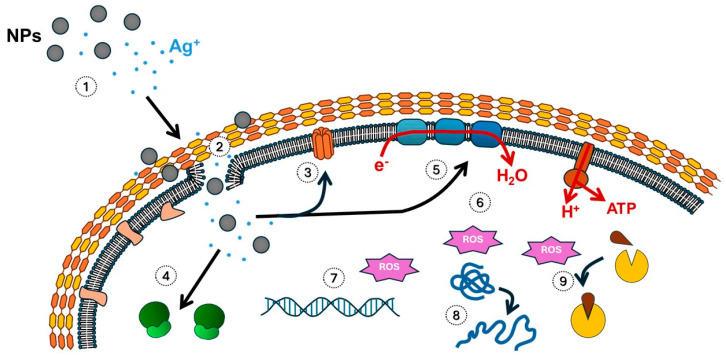
Mechanisms involved in the effect of AgNPs on bacteria. (1) The mechanism relies on the effect of the nanoparticles themselves and the release of silver ions (Ag^+^). (2) Their interaction with the cell envelope causes damage to the cell wall and destabilization of the membrane, resulting in ruptures and an increase in permeability, which facilitates the entry of AgNPs into the cell. (3) Their interaction with membrane proteins, such as efflux pumps, causes structural damage and contributes to membrane rupture. (4) AgNPs can denature ribosomes. (5) Inactivation of the respiratory chain, with inhibition of ATP production. (6) Formation of ROS. ROS can act by damaging nucleic acids (7), denaturing proteins (8), or causing enzyme disruption (9).

In order to undertake oxidative dissolution, AgNPs need an oxidizing agent like O_2_^−^ or H_2_O_2_, and this process releases Ag^+^, generating ROS inside cells, which accelerates the dissolution of AgNPs. The oxidation of Ag^0^ is one of the main mechanisms behind AgNPs’ antimicrobial effects, as the Ag^+^ interacts with microbial elements. Factors such as AgNPs’ size and surface coatings can impact the redox potential of Ag^0^ [28]. All these changes hinder bacterial growth and ultimately result in apoptosis [22,23,24,25,29] (Figure 2).

In particular, the excessive production of ROS leads to an oxidative imbalance that results in significant damage to lipids, proteins, and DNA. This oxidative stress triggers pro-apoptotic responses within the cell. A key consequence is the permeabilization of the outer mitochondrial membrane, which facilitates the translocation of cytochrome c from the mitochondrial intermembrane space into the cytosol. This process is tightly regulated by the differential expression of Bcl-2 family proteins, specifically the downregulation of anti-apoptotic Bcl-2 and upregulation of pro-apoptotic Bax, which, collectively, alter the mitochondrial membrane potential. The aforementioned mitochondrial perturbations initiate a cascade of caspase activation, producing a series of controlled events that culminate in cell death [30].

Furthermore, the elevated ROS levels activate p53, which a is a tumor suppressor that induces the expression of pro-apoptotic genes such as *Bax* and represses the PI3K/AKT/mTOR pathway, critical for cell proliferation. In cells such as HCT-116, AgNPs reduce AKT and mTOR phosphorylation, promoting apoptosis [31].

#### 3.1.2. Activity of AgNPs Against Fungus

Although it is noted that the antifungal effect of AgNPs depends on the size, shape, and dose of the nanomaterial, the mechanism of action remains a topic of investigation [34]. The literature indicates that AgNPs may disrupt the cell membrane due to the release of Ag^+^. The high-affinity copper transporter plays a crucial role in the uptake of silver. When Ag^+^ penetrates cell membranes, the amount of ROS increases, leading to apoptosis [32]. Simultaneously, the accumulation of Ag^+^ triggers an efflux of potassium ions, depleting almost all intracellular potassium levels. This process inhibits H^+^-ATPase. Since the mitochondrial copper transporter Pic2 has a higher affinity for Ag^+^ compared to copper ions, Ag^+^ enters the mitochondria, reducing copper levels and increasing silver levels. Consequently, cytochrome c oxidase, which depends on copper, malfunctions, thus diminishing the rate of cellular respiration [35].

AgNPs themselves may also cause effects in fungal membranes. As AgNPs are charged positively and fugal membranes are charged negatively, there are attractive electrostatic interactions between them, which facilitate the penetration of AgNPs in the intracellular environment [8]. Moreover, AgNPs can also cause structural damage, and produce ROS, facilitating the uncontrolled efflux of cytoplasmic components and initiating lipid peroxidation and protein denaturalization, all of which ends in cell death. The fungal wall matrix, formed by chitin, glucans, and proteins, is particularly susceptible to AgNPs, since they can bind to β-1,3-glucans and N-acetylglucosamine polymers, leading to damage to the integrity of the membrane [28,33].

Fungal metabolism is also affected. Ag^+^ modifies the tricarboxylic acid cycle, ergosterol synthesis, and lipid metabolism, causing significant alterations in cell membranes [36]. Furthermore, asexual reproduction of the fungus is inhibited, due to the emergence of abnormal hyphae and spore germination [34].

It has been found that certain antifungal drugs, like amphotericin B and fluconazole, can also interact with AgNPs and enhance their effect [35]. For example, AgNPs allow amphotericin B to penetrate cellular barriers, creating a synergistic effect between the activity of the drug and the effect of Ag^+^ [47]. Moreover, poly(methacrylic acid)-AgNPs have been described as having synergistic activity with fluconazole against *C. albicans* resistant to fluconazole, inhibiting germ tube formation [48].

#### 3.1.3. Activity of AgNPs Against Protozoa

Protozoan parasites such as *Leishmania*, *Plasmodium*, and *Trypanosoma* take control of host cells, promoting parasite replication and survival, and inducing pathogenic processes [49]. These protozoa can persist inside the host, causing chronic infections that are very difficult to eliminate [50].

AgNPs have shown activity against parasites, although there is limited information about their exact mechanism of action. Similarly to bacteria and fungi, the size, shape, and concentration of AgNPs influence their anti-parasitic activity [37]. For example, it has been noted that while both silver nanowires and silver nanorings can reduce the proliferation of *Acanthamoeba castellanii* trophozoites, the morphology of the former is more effective, and can also inhibit the germination of *A. castellanii* cysts [39].

Additionally, AgNPs release Ag^+^ ions that cause damage to the microorganism’s membrane, leading to ROS production and cell death [37]. Macrophages primarily generate high ROS levels to eliminate bacteria, fungi, parasites, and viruses. Some protozoa, like *Leishmania*, may evade this detoxification process by modulating certain signaling cascade components, allowing the parasites to survive. Conversely, ROS production oxidizes AgNPs, amplifying ROS levels and therefore eradicating the microorganisms [37]. It seems that AgNPs can also modify reproduction, causing DNA damage, as well as activating genes involved in cell death [38].

#### 3.1.4. Activity of AgNPs Against Viruses

Recent studies have reported that AgNPs are effective against a broad spectrum of viruses, inactivating them or even altering their binding to host cells. Several types of viruses, including DNA and RNA, both enveloped and non-enveloped, have been studied [40]. The factors influencing the antiviral properties of AgNPs are similar to those affecting their antibacterial effect, specifically size, shape, concentration [51], and functionalization.

Various strategies, such as the surface modification of AgNPs, coating AgNPs, or combining them with other elements, can be employed to prevent direct contact between metallic nanoparticles (NPs) and cells, thus reducing their potential cytotoxicity. Furthermore, these techniques may also affect the interaction between viruses and AgNPs. AgNPs that are functionalized, coated, or incorporated into a composite material can present antiviral efficacy, depending on the characteristics of the functionalization, the coating, or the composite material. For instance, NPs encapsulated in organic or inorganic materials like chitosan, collagen, or gelatin have demonstrated significant antiviral activity, with a prolonged effect due to the slow release of Ag^+^ [52].

The mode of action of AgNPs involves physical interactions with either the bound virus–host complex or the free viral particle. In this regard, AgNPs can obstruct binding with the host, prevent the penetration process, or inactivate the viral particle [44]. It appears that AgNPs have the ability to damage essential viral elements, such as glycoproteins of the viral envelope or capsid proteins [53]. AgNPs demonstrate inhibitory effects on early stages of viral replication, specifically by disrupting viral attachment to host cell receptors or interfering with cell penetration mechanisms (entry phase). The inhibition of viral binding happens through two different methods:

1. Temporary interactions between antiviral agents, like AgNPs, and viral surface ligands or host cell receptors, which sterically hinder virion–cell interactions
2. Competitive binding of AgNPs to cellular receptor sites, effectively clocking these regions and preventing viral adhesion [41].

Although less understood, some mechanisms may inhibit the late steps of viral replication [40]. This inhibition is rarely observed, and it cannot be considered the primary antiviral mechanism of AgNPs [52].

The viral structure has a huge impact on the antiviral mechanism of action of AgNPs.

Viruses have developed two principal mechanisms for cellular exit. On the one hand, enveloped viruses assimilate a lipid membrane derived from the host as they penetrate cell membranes. During this procedure, the viral envelope generally acquires one or more viral glycoproteins that interact with receptors on the target cell and promote the fusion of membranes, which is essential for maintaining the integrity of enveloped viruses and for viral penetration [54]. On the other hand, non-enveloped viruses exit host cells through the disruption of the plasma membrane, which typically happens through cell lysis. Subsequently, non-enveloped viruses target new cells either by breaching limiting membranes to access the cytoplasm, or by directly injecting their nucleic acids into the host cell [55].

The structural differences between enveloped and non-enveloped viruses significantly influence the antiviral efficacy AgNPs. These divergences have deep implications for key aspects of AgNP–virus interactions, particularly in regard to mechanisms of action, the cytotoxic relationship between AgNP concentration and host cells, and AgNPs’ surface coating [42,56,57].

AgNPs present different mechanisms of action against enveloped viruses. AgNPs can bind to sulfur-rich domains of surface glycoproteins, like hemagglutinin and amidasa in influenza, blocking receptor-mediated host cell entry. In this case, the size of the particle is particularly important, due to the fact that the smaller the AgNP is, the better its binding to proteins [40,42,53]. Moreover, AgNPs can produce ROS and denature the lipid envelope.

In regard to non-enveloped viruses, AgNPs can adhere to the surface of viral particles, reducing the possibility of aggregation [46]. Furthermore, AgNPs can penetrate these types of viruses and cause damage to their nucleic acids [44,45]. Eventually, this nanomaterial is able to interact and disrupt the capsid proteins [58].

The enhanced stability of non-enveloped viruses necessitates higher concentrations of AgNPs for effective viral inhibition. This requirement for huge AgNP dosage significantly elevates the risk of cytotoxicity to host cells. The need to balance efficacy against non-enveloped viruses with the potential for cellular damage presents a considerable challenge in the application of AgNPs as antiviral agents in these cases [57].

Coated AgNPs have been observed to confer greater benefits, mitigating the cytotoxicity associated with AgNPs and demonstrating variations in their antiviral efficacy against both enveloped and non-enveloped viruses. For instance, AgNPs that are coated with polyvinylpyrrolidone have been shown to exhibit remarkable antiviral properties against enveloped viruses, including respiratory syncytial virus and human immunodeficiency virus [56]. Another example could be the antiviral efficacy of AgNPs coated with graphene oxide sheets. Their effect against enveloped viruses may be ascribed to a significant physicochemical interaction between AgNPs and the lipid components of the viral envelope. In regard to non-enveloped viruses, coating AgNPs with graphene oxide sheets inhibits agglomerate formation, and enhances the antiviral properties of silver [40].

### 3.2. AgNPs to Combat Antibiotic Resistance

In recent years, the emergence of bacterial resistance to antibiotics has gradually increased, making bacterial infections increasingly difficult to eliminate. It is projected that by 2050, antibiotic-resistant bacteria will cause 10 million deaths annually [59]. The overuse of antibiotics in medical treatments, inadequate dosing, and excessive prescriptions in farming and agriculture have led to the uncontrolled proliferation of antibiotic resistance. Notably, *Staphylococcus aureus*, *Pseudomonas aeruginosa*, and *Enterococcus faecium* are regarded as multidrug-resistant strains [60]. Given the rapid spread of this epidemic, new strategies to combat infections are urgently needed [61].

Efflux pumps play a crucial role in removing harmful compounds from intracellular environments, as they transport toxic chemicals and antibiotics out of the cell before the drugs can exert their effects [62].

Other mechanisms involved in developing antibiotic resistance include altered cell permeability, changes in the antibiotic target site, enzymatic inhibition of antibiotics, and the synthesis of new enzymes that do not bind to antibiotics [63].

Recently, nanoparticles (NPs) have emerged as a novel and promising tool, offering not only localized therapeutic effects, but also targeted intracellular delivery. Certain excipients can hinder efflux pump activity [64]. NPs act as delivery vehicles, intensifying drug concentration within bacterial cells. Additionally, the combined administration of NPs and efflux transporter inhibitors has the potential to improve the therapeutic efficacy of antibiotics against resistant bacterial strains [64].

For instance, combinations of antibiotics such as ciprofloxacin, vancomycin, trimethoprim, and gentamycin with AgNPs have been studied to enhance drug potency. The results showed an increase in antibacterial properties, with the combination of antibiotics and AgNPs delaying the development of resistance by reducing the amount of antibiotics needed for treatment [24]. For instance, the synergistic interactions of AgNPs with aminoglycoside antibiotics, evidenced by a 22-fold reduction in the minimum inhibitory concentration of amikacin, indicate significant potential for the application of AgNPs as adjuncts to antibiotic therapy [65].

Interestingly, AgNPs can reverse bacterial resistance to streptomycin. Mutations in the *rpsL* gene, which encodes the ribosomal S12 polypeptide, can modify this protein, altering its interaction with streptomycin and eventually leading to bacterial resistance. AgNPs have been demonstrated to increase mutation frequency by binding to DNA, potentially countering this resistance [46].

Signal transduction mechanisms in bacteria represent a significant focus of the antibacterial efficacy of AgNPs. Microbial communities demonstrate remarkable sensitivity and adaptive responses to diverse environmental stimuli. These capabilities are mediated through signal transduction systems that regulate bacterial persistence, population dynamics, and virulence factor expression. These signaling systems have consequently emerged as critical targets for developing next-generation antimicrobial strategies [66]. AgNPs in the 10–15 nm size range can act as potent modulators of bacterial signaling pathways. Their mechanism of action involves targeted disruption of tyrosine residue phosphorylation kinetics on essential signaling proteins, which subsequently triggers programmed cell death or growth arrest in microbial populations [67].

Caution is paramount in the use of AgNPs against bacteria. The dissemination of antibiotic resistance genes predominantly happens through horizontal gene transfer, a critical mechanism driving antimicrobial resistance proliferation. Emerging evidence indicates that AgNPs exacerbate this process by enhancing the transfer of plasmid-mediated antibiotic resistance genes [68]. It seems that long-term exposure to sub-lethal concentrations of AgNPs can lead to the development of resistance [69]. For instance, proteomic analysis has shown that certain transcriptional factors, such as Lux and Rex, and enzymes related to oxidative stress, like superoxide dismutase, change after the interaction between *E. faecalis* and AgNPs [70].

Furthermore, bacteria can enhance antibiotic resistance through biofilm formation [71]. Biofilms provide a protective environment that increases bacterial resistance to antibiotics. AgNPs have shown potential in disrupting biofilms, thus improving the efficacy of antibiotic treatments [72].

### 3.3. Anti-Biofilm Activity of AgNPs

Bacteria have the ability to form structures called biofilms, which are a major cause of chronic infections. These biofilms are responsible for over 50% of nosocomial infections, and can be found on medical equipment such as catheters, cardiac pacemakers, and heart valves [72]. Biofilms are complex and dynamic systems composed of single or multiple bacterial strains, archaea, fungi, and protozoa embedded in an extracellular polymeric substance (EPS) [59,72,73]. The EPS facilitates bacterial interactions, nutrient transport, and attachment to various surfaces. It primarily consists of water and proteins, with smaller amounts of DNA, RNA, ions, polysaccharides, and inorganic substances [74]. DNA in the EPS protects the biofilm by facilitating horizontal gene transfer, which is crucial for developing resistance against antibiotics [22].

Biofilms provide several advantages to microorganisms over their planktonic state, including protection against osmotic stress, pH changes, nutrient scarcity, and immune system phagocytosis [75]. While our immune system can eliminate planktonic bacteria easily, bacteria within biofilms can survive immune defenses and antibiotics, leading to recurrent infections [76].

The development of biofilms involves several stages. Initially, planktonic microorganisms adhere to surfaces, a process that can be reversible or irreversible. Subsequently, microorganisms proliferate and mature within the biofilm structure. Finally, planktonic cells disperse to seek new colonization sites and form new biofilms, perpetuating the cycle indefinitely [77]. Therefore, a mature biofilm contains sessile (attached), planktonic (free-floating), persistent (tolerant), and dead cells, as well as water channels, signaling molecules, and all components of the EPS [78].

The anti-biofilm activity of AgNPs has been extensively studied against both Gram-positive and Gram-negative bacteria [79]. In these systems, AgNPs are capable of eliminating bacteria through mechanisms similar to those observed in planktonic microorganisms [80].

AgNPs and Ag^+^ ions are able to interact with various components of the EPS, such as DNA, RNA, proteins, lipids, and polysaccharides. Regarding RNA, there is evidence suggesting an association between AgNPs and small regulatory RNAs, altering RNA expression and suppressing biofilm formation [22].

Quorum sensing is crucial in order for bacteria to regulate gene expression in response to changes in population density. Bacteria produce and release signaling molecules called autoinducers, which accumulate at high concentrations with increasing cell density. Upon reaching a specific threshold concentration, these autoinducers trigger changes in gene expression [81]. Both Gram-positive and Gram-negative bacteria use quorum sensing to regulate various physiological processes, including the coordination of biofilm development [82]. AgNPs interact with proteins involved in quorum sensing, particularly amino acids in binding regions, thereby deactivating these sites and disrupting cell signaling. AgNPs also impact bacterial structure by interacting with amyloid-forming proteins through hydrophobic and electrostatic forces, as well as with metabolic proteins and protein oligomers, leading to their inactivation [22].

Lipids play a critical role in adhesion to surfaces and biofilm formation [83]. AgNPs primarily interact with lipids through hydrophobic and electrostatic interactions. AgNPs disrupt membrane fluidity via hydrophobic interactions, destabilizing the membrane and ultimately leading to bacterial cell elimination [22].

Biofilm structure consists of two types of polysaccharides: those associated with bacterial cell walls and those in the extracellular environment. Cell wall-associated polysaccharides provide stability, osmoregulation, mechanical strength, and virulence, while extracellular polysaccharides are involved in functions such as adhesion, cohesion, the formation of a protective barrier, the maintenance of biofilm integrity, the promotion of interactions between bacteria, and the preservation of nutrient sources within the biofilm [84]. AgNPs interact with both types of polysaccharides, establishing electrostatic forces with cell wall-associated polysaccharides to enhance toxicity toward bacteria. The interaction between AgNPs and extracellular polysaccharides remains understudied, but it is known that AgNPs can hinder access to the biofilm matrix [22].

In biological environments, peptides, amino acids, and proteins adhere to NP surfaces, forming an NP corona. These biomolecules can alter interactions between NPs and EPS components, complicating the study of exact AgNP–EPS interactions [22].

It is undoubtable that Gram-positive biofilms are more susceptible to the influence of AgNPs compared to their Gram-negative counterparts. This fact is mainly due to the composition of the Gram-positive cell wall. Certain components, such as lipoteichoic and teichoic acids, give Gram-positives a more pronounced electrical charge in comparison to Gram-negatives. Since AgNPs possess a positive electrical charge, the electrostatic interaction between AgNPs and Gram-positives is better compared with the interaction between AgNPs and Gram-negatives [85].

Certain environmental factors must be considered in regard to the efficacy of AgNPs in combating biofilms. For instance, low phosphorous levels increase exopolysaccharide sulfation, which leads to enhanced metal ion chelation [86]. Temperature shifts produce changes in *luxS* expression, which cause variations in quorum sensing effectiveness [87], and oxygen gradients cause the presence of stratified metabolic areas, which encourage polymicrobial synergy [88].

**Figure 3:**
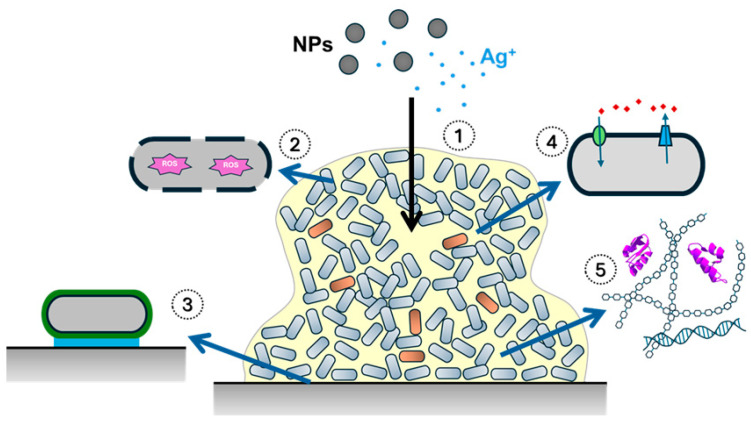
Mechanisms involved in the effect of AgNPs on biofilms. (1) The mechanisms are based on the effect of the nanoparticles themselves and the release of silver ions (Ag^+^). (2) AgNPs can act on bacteria using the same mechanisms as against planktonic cells. (3) The destabilization of cell envelopes by AgNPs prevents adhesion to surfaces. (4) AgNPs interact with proteins involved in quorum sensing, disrupting cell signaling. (5) AgNPs can interact with different components of the extracellular polymeric substance, including nucleic acids, proteins, and polysaccharides, destabilizing it. The presence of a small percentage of persistent cells (orange color) is essential for bacterial survival, although the effect of AgNPs on them is unknown.

In biofilms, some bacterial populations reside in nutrient- and oxygen-depleted areas, reducing their metabolic activity and increasing their division times. These subsets of bacterial populations, known as persister cells, are crucial in developing resistance to antibiotics. Moreover, certain mechanisms of resistance, such as mutations in antibiotic targets or upregulated efflux pumps, strengthen biofilm structure [89]. Persister cells constitute a small percentage of the bacterial population, and can tolerate the presence of antibiotics, resuming growth when conditions become favorable. They are also referred to as small-colony variants, with their extraordinary tolerance attributed to low metabolic rates and slow growth [90]. To the best of the authors’ knowledge, there is currently no information available on the use of AgNPs to specifically target persister cells (Figure 3).

The effect of sub-lethal concentrations of AgNPs has also been studied [91]. Research indicates that these nanostructures can stimulate biofilm formation at low concentrations, acting as environmental stressors that trigger defense mechanisms such as the upregulation of quorum sensing and the biosynthesis of sugars, lipopolysaccharides, and other protein components of EPSs [92].

AgNPs have demonstrated antibacterial activity against biofilms composed of multidrug-resistant strains. For instance, combining AgNPs with traditional antimicrobials like rifampicin significantly enhances antibiofilm properties, reducing biofilm formation by up to 90% with lower concentrations of AgNPs. Thus, nanoconjugates improve penetration of conventional antibiotics into biofilms [93].

Despite their effectiveness in inhibiting biofilm growth, scientific evidence shows that bacteria can develop tolerance to AgNPs after prolonged exposure to nanosilver. For example, *Staphylococcus aureus* can undergo genetic mutations to enhance resistance characteristics, even when AgNPs are no longer in direct contact with the bacteria [94]. While AgNPs are effective in inhibiting biofilm growth, there are findings suggesting that this nanomaterial may not be effective against established biofilm biomass. Therefore, AgNPs should not be used for prolonged treatments against biofilm infections [72].

Recent findings have acknowledged that bacteria employ some strategies to tolerate the presence of AgNPs. Aggregation and the dissolution state play a key role in AgNPs’ effectiveness. When AgNPs are aggregated, they release less Ag^+^, decreasing their antibacterial activity. In contrast, when the amount of AgNPs rises, they release more Ag^+^, so their antibacterial activity increases [95]. The production of flagellin, as a stabilizing agent, improves the bacterial tolerance to AgNPs. This is the case of *E. coli* and *P. aeruginosa*, although it seems that other bacterial components, such as the EPS or the ribosomal proteins, can also help bacteria to adapt to the presence of AgNPs. Other strategies are the bacterial production of reducing agents, like pyocianin, that reduce Ag^+^ to Ag^0^, and the transformation of AgNPs or Ag^+^ to Ag_2_S [96].

Antibiotic resistance may also confer resistance to silver-based antimicrobial agents. AgNPs and Ag^+^ have the ability to disrupt established biofilms of *P.aeruginosa* at certain concentrations. Nevertheless, both AgNPs and Ag^+^ at identical concentrations failed to eliminate the biofilm of mature gentamicin-resistant *Pseudomonas aeruginosa* [72]. The mechanism of resistance may be attributed to AgNPs’ blocked penetration, bacterial aggregation within biofilms, and an increase in intercellular adhesion [96]. Other bacteria, such as methicillin-resistant *S. aureus*, ampicillin-resistant *E. coli*, ampicillin-resistant *Klebsiella pneumoniae, Acinetobacter baumanii*, and *Salmonella typhi*, have also shown resistance to AgNPs [97].

Eventually, some bacteria can develop resistance to AgNPs due to the possibility of mutating resistance genes [68]. A study of *E. faecalis* demonstrated the ability of these microorganisms to develop resistance against AgNPs through the oxidative stress response, transcriptional regulatory factors, universal stress proteins, and fibronectin adhesin. To prevent the emergence of resistance to AgNPs, the study proposed the simultaneous application of AgNPs derived from different metals to hinder the spread of resistant strains [70]. The lateral transfer of antibiotic resistance genes is acknowledged as one of the main methods of resistance propagation, and it seems that AgNPs promote the transfer of antibiotic resistance genes via plasmid transmission among various bacteria [68].

## 4. AgNPs in Non-Infectious Pathologies

As previously mentioned, silver nanoparticles (AgNPs) not only represent a promising strategy for treating infections, but also find applications in various other medical fields, such as cancer therapy, diabetes treatment, and wound healing [98,99,100].

In order to facilitate the reader’s understanding, a table with the therapeutic properties and mechanisms of action of AgNPs related to non-infectious pathologies, along with references, is included below (Table 3).

### 4.1. Diabetes

Diabetes affects approximately 422 million people worldwide, and is responsible for 1.6 million deaths annually [100]. It is characterized by chronic hyperglycemia, which progressively damages organs such as the eyes, nerves, kidneys, heart, and blood vessels [121]. The primary causes of hyperglycemia in diabetes include reduced insulin production (type I diabetes) or inefficient insulin utilization (type II diabetes) [101].

One of the key factors influencing diabetes development is the production of ROS, triggered by glucose and fatty acid overload. Oxidative stress leads to pancreatic cell death, contributing to insulin depletion in diabetes. Managing diabetes involves reducing ROS production to mitigate oxidative stress and prevent cell death [103].

AgNPs have shown potential anti-diabetic properties by influencing ROS levels. Studies indicate that AgNPs can regulate glucose and insulin levels, reduce inflammation, and inhibit apoptosis in pancreatic cells in murine models. Additionally, AgNPs contribute to restoring normal histology in organs like the kidneys and liver after administration in animal models [104].

Pancreatic α-amylase is responsible for the breakdown of dietary carbohydrates into monosaccharides within the digestive system. Subsequently, these monosaccharides are degraded by α-glucosidases to glucose, which can enter the bloodstream. Hence, α-amylase and α-glucosidase inhibitors prevent carbohydrate digestion, leading to a delay in glucose absorption, and thereby reducing blood glucose levels [105]. This is why α-amylase and α-glucosidase inhibitors play a significant role in the treatment of diabetes and glucose intolerance [106].

**Figure 4:**
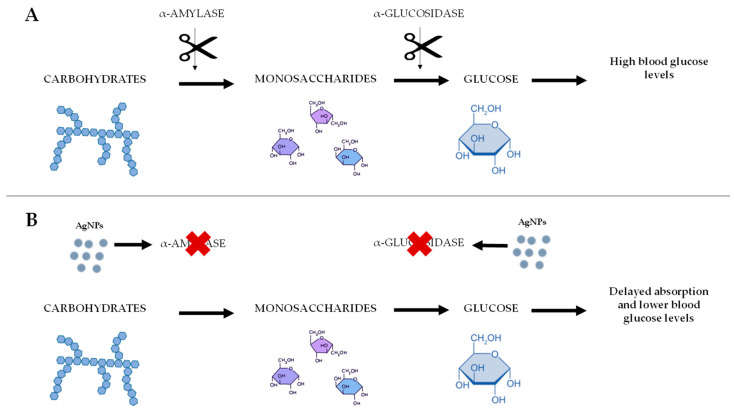
Sequential process of carbohydrate digestion, enzyme inhibition by AgNPs, and resulting therapeutic benefits for glucose regulation. (**A**) Sequential process of carbohydrate digestion. (**B**) Process of carbohydrate digestion after AgNP interaction.

AgNPs have been identified as inhibitors of α-amylase and α-glucosidase enzymes, thereby reducing blood sugar levels [107,108]. They can bind to the active sites of α-amylase, preventing the enzyme from catalyzing carbohydrate hydrolysis. This inhibition reduces carbohydrate digestion and subsequent glucose release, moderating spikes in postprandial blood glucose levels [110] (Figure 4).

Moreover, AgNPs have been reported to exhibit anti-diabetic properties by enhancing the activation of GLUT2 (hepatic glucose transporter 2 gene), increasing serum insulin levels, and enhancing liver glucokinase activity [102]. AgNPs can elevate intracellular calcium ion concentrations, facilitating the activation of AMP-activated protein kinase (AMPK). AMPK plays a critical role in regulating cellular energy status in eukaryotic cells. Activation of AMPK enhances insulin responsiveness, promoting glucose transport into cells. AgNPs have been associated with decreased blood glucose levels by increasing expression levels of IRS1 (insulin receptor substrate 1) and glucose transporter 2 [109].

To sum up, AgNPs show promise in diabetes management through their ability to regulate glucose metabolism, inhibit digestive enzymes involved in glucose absorption, reduce oxidative stress, and enhance insulin sensitivity, making them a potential therapeutic option for diabetes treatment.

### 4.2. Wound Healing

A wound is defined as any disruption in anatomical structure or function that results in a break in the integrity of the skin. Wounds are sometimes referred to as “silent epidemics” due to the severe consequences of untreated cases, including potential amputations or even death. Approximately 8.2 million patients suffer from chronic and incurable wounds [122]. Both diabetic and non-diabetic individuals face significant challenges in wound healing and prevention. Therefore, there is a critical need to explore new approaches to enhance the wound healing process [15].

The remarkable anti-inflammatory and antibacterial properties of silver nanoparticles (AgNPs) have led to their use in wound healing therapies. AgNPs are applied to create a moist environment around the wound, which aids in promoting sustained oxygen delivery during the healing phase. Additionally, AgNPs prevent microbial growth around the wound, due to their broad-spectrum antibacterial activity [110].

AgNPs exhibit anti-inflammatory properties by modulating the immune response against foreign particles. They have been shown to reduce the expression of inflammatory cytokines such as tumor necrosis factor α and transforming growth factor α. When topically applied to the wound area, AgNPs decrease the release of cytokines and lymphocytes and reduce mast cell infiltration, thereby facilitating wound repair with minimal scarring [111].

Studies indicate that AgNPs accelerate wound closure by enhancing the proliferation and migration of keratinocytes within the wound area. Moreover, AgNPs stimulate the transformation of fibroblasts into myofibroblasts, thereby supporting wound contraction. It has also been demonstrated that AgNPs regulate collagen deposition and alignment. Ultimately, AgNPs promote re-epithelialization and differentiation of fibroblasts, contributing to efficient wound healing [112].

In summary, AgNPs play a significant role in wound healing processes by creating an optimal environment for healing, preventing infections, reducing inflammation, and promoting tissue regeneration. Their multifaceted properties make them a promising candidate for improving therapeutic outcomes in wound care.

### 4.3. Cancer

Currently, cancer is recognized as the second leading cause of mortality globally. Cancer encompasses a group of diseases characterized by rapid cell proliferation and alterations in various physiological cellular processes. Surgery, chemotherapy, and radiotherapy are presently the predominant cancer treatments, despite their potential side effects, the development of resistance, and complications such as incomplete tumor removal. Consequently, nanomedicine applications in oncology have been extensively researched. Cancer nanomedicine aims to achieve early tumor detection, precise diagnosis, and personalized therapies [123].

#### 4.3.1. Antitumor Properties of AgNPs

The antitumor effects of silver nanoparticles (AgNPs) depend on several factors, including the specific type of affected cell and the size and shape of the nanomaterial [13]. The precise mechanisms by which AgNPs act on tumors are not fully elucidated, but it is acknowledged that ROS play a role in their cytotoxic effects [113]. ROS can disrupt mitochondrial structure and function, donating electrons to oxygen to generate superoxide anions (O_2_^−^). Normally, cells maintain low levels of superoxide anions, as well as hydroxyl radicals and hydrogen peroxide, through their antioxidant defense systems, as these molecules also function in cellular signaling pathways. However, increased ROS levels lead to oxidative stress. AgNPs elevate ROS concentrations, inducing oxidative stress that inhibits cell proliferation and damages macromolecules and organelles, ultimately leading to cell death [114].

**Figure 5:**
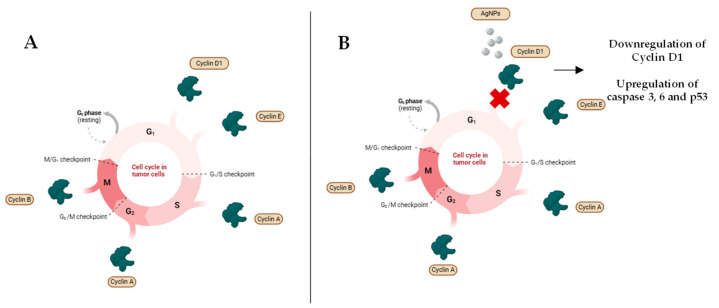
Cell cycle in tumor cells, before and after interaction with AgNPs. (**A**) Cell cycle in tumor cells in standard conditions. (**B**) Cell cycle in tumor cells after interaction with AgNPs. Created with BioRender.com.

AgNPs can also affect processes involved in the cell cycle of tumor cells. Cyclin D1 and cyclin E are critical in transitioning from the G1 to S phase. Interaction between lung carcinoma cells and AgNPs appears to arrest cells in the G1 phase and promote apoptosis by downregulating Cyclin D1 and upregulating caspase 3, 9, and p53. Thus, AgNPs can delay tumor progression and inhibit cell proliferation [13,117] (Figure 5).

Nanotechnology can be utilized to target other mechanisms involved in cancer, such as angiogenesis and metastasis [115]. Angiogenesis, the formation of new blood vessels [116], is often dysregulated in tumors, due to elevated levels of angiogenic stimulators like tumor angiogenesis factor, which are secreted by tumors in response to nutrient and oxygen demands, promoting tumor growth. Angiogenesis also facilitates metastasis, the process by which tumor cells infiltrate the vascular system, migrate to distant sites, and proliferate. AgNPs have shown the ability to suppress genes associated with angiogenesis, such as vascular endothelial growth factor, and deactivate related signaling pathways [115].

Although AgNPs have shown huge potential in cancer therapy due to their cytotoxic effects on tumor cells, their off-target toxicity remains a critical concern, as they may also have an effect on healthy cells. As it has been mentioned before, AgNPs can cause oxidative stress, apoptosis, and cell cycle interference, but something that must be faced is the fact that these mechanisms affect both healthy and tumor cells [124].

A significant limitation of the use of AgNPs for tumoral cells target lies in the absence of a robust tumor-specific delivery mechanism. Recent studies have highlighted exosomes as innovative nanoscale delivery platforms, due to their stability, biocompatibility, biodegradability, and capacity to target tumors [118]. These extracellular vesicles are released by healthy and pathological cells, and play a key role in intercellular communication by cell signaling and transferring their content through a new cell [125]. AgNPs and copper nanoparticles have been introduced in exosomes during their formation in healthy cells, protecting this nanomaterial from acid environments and demonstrating selective cytotoxicity against breast tumoral cells [118].

Surface functionalization and antibody-targeted delivery are another two strategies to mitigate off-target cytotoxicity. For instance, AgNPs functionalized with glutamine and conjugated with thiosemiccarbazide were shown to be more cytotoxic to colon tumor cells than to healthy fibroblasts [119], and a mix of AgNPs and anti-CD20 antibody Rituximab was able to increase the cytotoxicity and specificity of AgNPs against chronic lymphocytic leukemia cells [120].

#### 4.3.2. Combination of AgNPs with Chemotherapy and Radiotherapy

As previously mentioned, chemotherapy remains one of the primary treatments for cancer. Despite its efficacy against various tumor types, chemotherapy is hindered by significant limitations, such as high side effects, poor water solubility, limited ability to penetrate tumor cells, non-selective targeting, and the development of chemotherapy resistance [126].

Combination chemotherapy involves using two or more drugs to target multiple cancer characteristics simultaneously, aiming to produce a synergistic effect. This approach has the potential to enhance effectiveness, reduce drug resistance, and mitigate side effects [127]. However, adjusting dosages and overcoming differences in the pharmacokinetic and physicochemical properties of the drugs is challenging. Silver nanoparticles (AgNPs) show promise in synergizing with chemotherapeutic agents by facilitating drug delivery into target cells, controlled release of drugs, addressing solubility challenges, and enhancing the toxicity of anticancer agents like cisplatin or methotrexate.

AgNPs can also be combined with radiotherapy, as these nanomaterials can act as radiosensitizers. They release electrons that augment the damage caused by radiation within cells. Additionally, AgNPs may reduce cell proliferation and increase autophagy and apoptosis [114].

## 5. Biosensing

The primary objective of biosensors is to measure the biological components present in a given sample. These devices convert physicochemical changes into measurable signals to detect specific analytes of interest. Biosensors can identify various analytes, such as DNA, mRNA, enzymes, and small molecules [128].

Nanosensors consist of a nanomaterial that detects a parameter and a physical transducer. The analyte of interest could be a pharmaceutically active substance or a biomolecular analyte [129].

Metal nanoparticles (NPs) are versatile, relatively cost-effective, and provide a rapid platform for signal amplification. They can detect biomarkers in real-time, with a low limit of detection, and are user-friendly [129].

Due to their substantial surface area and excellent biocompatibility, AgNPs can immobilize a large number of biomarkers on their surface. Additionally, these nanomaterials have the ability to label biological components, while preserving their biological characteristics [130].

Biosensors can be classified into four different categories: DNA biosensors, enzyme biosensors, whole-cell biosensors, and phage biosensors, depending on the specific type of bioreceptor employed. Moreover, based on the various types of transducers utilized, a biosensor may be categorized as a piezoelectric biosensor, an electrochemical biosensor, or an optical biosensors [131]. Piezoelectric biosensors are based on the measurement of changes in resonance frequency [132].

In regard to electrochemical biosensors, the electrochemical properties of AgNPs make them highly suitable for use in biosensors employing various transduction techniques [129].

Electrochemical bioassays are crucial in diagnostic applications, due to their rapid response, affordability, and ease of use. Having stable and robust electrochemical labels is paramount in these assays, especially when the analyte lacks inherent electrochemical activity. While enzymes are commonly used as labels, they have limitations such as fragility and sensitivity to pH and temperature. AgNPs offer a promising alternative to enzymatic labels by mimicking the amplification role of enzymes and providing faster sample-to-answer times [71].

In the field of electrochemical tags, AgNPs can specifically identify tumor protein biomarkers. This approach is cost-effective, rapid, and flexible compared to traditional ELISA methods. Moreover, it enables the detection of multiple targets, thereby enhancing the precision of cancer diagnosis. Point-of-care tests, tumor phenotyping, and liquid biopsy could benefit significantly from this approach [133].

AgNPs have demonstrated a range of unique optical characteristics that can be effectively utilized for detection purposes by transducers frequently employed within biosensor technology. The freely mobile electrons within these AgNPs, upon excitation by visible wavelengths, induce surface plasmon resonance, which subsequently results in a pronounced absorption phenomenon that depends on the shape, size, and dispersion of the AgNPs [129].

Recently, there has been increasing interest in nanoplasmonic biosensors, due to their distinctive optical properties and precise manipulability. Nanoplasmonic biosensors rely on localized surface plasmon resonance (LSPR), where incident light induces electron oscillations in NPs at wavelengths equal to or shorter than the incident light wavelength [134]. Biosensors utilizing LSPR technology employ metals such as gold and silver to achieve highly sensitive detection of target molecules in medical applications [135].

These biosensors find broad applications in medicine by detecting antigens and microbes, and quantifying the affinities and kinetics of biomolecular interactions. LSPR sensors are particularly useful in point-of-care settings and healthcare monitoring, including for the detection of explosives, heavy metals, and gases [134].

Biosensors can also detect changes in optical characteristics, such as luminescence, polarization, and absorption, due to their interaction with specific analytes [136]. For instance, biosensors can count the total number of bacteria in a sample without requiring an enrichment procedure, which is highly beneficial for diagnostic and quantitative purposes [137].

## 6. Toxicity of AgNPs for the Environment and Human Beings

NPs are considered an emerging type of environmental contaminant characterized by distinct physicochemical properties compared to those of conventional pollutants. These attributes, including an elevated surface area-to-volume ratio and enhanced chemical reactivity, allow NPs to engage with biological systems through novel mechanisms that may pose toxicological risks [138].

In aquatic ecosystems, NP exposure pathways include dermal uptake, aqueous ingestion, or adsorption onto trophic transfer vectors [138]. The subsequent vascular dissemination facilitates systemic bioaccumulation, with preferential deposition observed in the bones, liver, and kidneys as well [139]. Specifically, AgNPs can induce unwelcome effects in aquatic organisms, such as modifications to the organisms’ antioxidant defense mechanisms, feeding patterns, reproductive abilities, metabolic functions, growth rates, and maturation. In severe cases, exposure to AgNPs may end in mortality [140]. Moreover, NPs exhibit the potential to biomagnificate their effects, leading to an amplification of toxicity from lower to higher trophic levels [141,142].

Hence, AgNPs can be a hazard to animals, plants, and humans. The literature indicates that AgNPs exert pronounced effects on plant physiological parameters, change the structural composition of soil microbial communities, and modify soil enzymatic function [143]. AgNPs demonstrate preferential bioaccumulation in plant tissues, particularly in root systems. Notably, AgNP exposure exhibits stimulatory effects on root system elongation across multiple agriculturally significant plant species [144].

Different oxidoreductase enzymes, like catalase or peroxidases, play a key role in environmental bioremediation, organic matter decomposition, and microbial metabolic regulation [144]. Catalase activity exhibits dose-dependent reductions when exposed to environmental pollutants, particularly under conditions of petroleum hydrocarbon and heavy metal contamination [145].

AgNPs are considered risky due to their ability to generate ROS, cause DNA damage, and induce inflammation. High ROS levels can lead to harmful effects if they are not properly regulated [146]. The toxicity of AgNPs is influenced by factors such as their release of Ag^+^ ions, coatings, formulation, and particle size [147].

It is crystal clear that AgNPs have the potential to cause damage to various organs and systems in the body, including the respiratory, gastrointestinal, and reproductive systems [148]. Many in vivo and in vitro studies have been published regarding the potential toxic effect of AgNPs in animals and humans.

In vitro experiments have shown that AgNPs produce ROS, and, therefore, genotoxic stress in lung epithelial cell lines (A549 and BEAS-2B), triggering DNA strand breaks and, finally, apoptosis [149]. In vitro lymphocyte cultures exposed to AgNPs exhibited a rise in micronucleus generation after 24 h and 48 h of exposure. Likewise, this nanomaterial diminishes the cytokinesis-block proliferation index and mitotic index at the same times of exposure [150]. In human umbilical vein endothelial cells, AgNPs demonstrated high pro-apoptotic activity, anti-proliferative effects, and membrane integrity compromise [151]. In embryonic neural stem cells derived from both humans and rats, exposure to AgNPs averaging 23 nm in diameter resulted in a pronounced reduction in cellular proliferation, which can be ascribed to excessive generation of ROS and lactate dehydrogenase [152].

In regard to in vivo studies, it has been found that kidneys, heart, and liver are affected in male rats after exposure to AgNPs. Hepatic tissues manifest oxidative stress, inflammatory responses, and compromised detoxification capacity, which are indicative of metabolic dysregulation. The renal system suffers nephrotoxicity, due to perturbations in glomerular filtration rates, tubular dysfunction, and histopathological alterations such as interstitial fibrosis. Furthermore, cardiomyocytes in the heart cause oxidative damage cardiac tissues, and the myocardial structure is affected by necrosis and inflammatory infiltration [153].

AgNPs also have the potential to accumulate in fish, producing histopathological alterations in gill, hepatic, and renal tissues. AgNPs disrupt critical physiological mechanisms through oxidative stress induction, osmoregulatory imbalance, and homeostasis dysregulation, which are related to behavioral anomalies, developmental reduction, and diminished reproductive ability [154].

Tadpoles of *Polypedates maculatus* suffered significant alterations in their hematological system, metabolic homeostasis, and immune competence [139], and *Culicoides sonorensis* exhibited neuromuscular disruption, and even death, after 24 h of exposure [155].

AgNPs have been implicated in pathological processes affecting both male and female reproductive systems [101]. Studies have shown that AgNPs can interfere with ovarian function and alter energy balance in targeted cells in chicken ovaries [103]. Furthermore, AgNPs have the potential to significantly impact male fertility in rats, leading to degenerative changes in testicular and epididymal cellular structure [104].

These evidences show that AgNPs are able to be transferred through different trophic levels, with the possibility of ultimately affecting human populations [156]. The main pathways of human exposure to AgNPs are oral, respiratory, and dermal routes [145].

Oral exposure to AgNPs has been documented through the consumption of contaminated food. It has been observed that AgNPs can alter gut microbiota after prolonged oral administration, although bacteria can recover due to their resilience. Nonetheless, significant changes in gut microbiota metabolites have been noted [157].

The respiratory system is a primary route of exposure to NPs. Lung cells can internalize AgNPs, leading to the dissolution and release of high concentrations of ions. Both AgNPs and Ag^+^ ions can enter mitochondria or nuclei, causing oxidative damage. Larger AgNPs may not be internalized through endocytosis, but have shown cytotoxic activity via receptor-mediated transduction routes [147].

The skin, the body’s largest organ and main barrier against environmental factors, represents a significant route of human contact with AgNPs. This nanomaterial is able to traverse intact and compromised dermal layers, penetrating more deeply in different tissues. This fact has raised some concerns, since it seems that the cosmetic industry uses AgNPs in up to 20% of product formulations [158].

However, considering all the information above, it is imperative to critically evaluate the potential toxicity associated with conventional antimicrobial agents, and compare these data with the toxicity profile of AgNPs. A side-by-side quantitative comparison of the toxicity of AgNPs versus traditional antimicrobials is difficult to make, as therapeutic drug monitoring is less studied for AgNPs compared to traditional antimicrobial agents. However, some key aspects that enable this head-to-head comparison are discussed below:

- Specificity: Traditional antimicrobials, while designed to disrupt specific bacterial processes, can also damage the beneficial microbiota in the body. This disruption can lead to the proliferation of already-present harmful bacteria, the overgrowth of pathogens with acquired resistance, and ultimately, a higher risk of infection [159]. AgNPs, while having broad-spectrum antimicrobial activity, can also induce oxidative stress and affect healthy cells [160].
  The off-target toxicity of AgNPs in biological systems has been extensively documented in recent studies [161,162,163,164]. It has been shown that AgNPs disrupt tight junction integrity (particularly occludin and zonulin proteins) in epithelial tissues, compromising epithelial barrier functionality [124]. AgNPs have been shown to alter the regulation of gene expression associated with motor neuron pathologies, neurodegenerative conditions, and immune cell functionality. For instance, they can be toxic to macrophages and monocytes [151,165]. Furthermore, AgNPs significantly modify cardiomyocytes’ contractility through interference with ion channel function and the disruption of key structural proteins [166].
  In some studies, the effect of repeated low-dose exposure to AgNPs has been studied. Other investigations have been conducted using high doses and short exposure durations. From an exposure perspective, acute protocols more closely simulate accidental and acute exposure scenarios, whereas chronic regimens largely correspond to occupational exposure scenarios [165,167,168].
  In addition, some strategies have been developed to reduce this off-target toxicity. Surface functionalization, green synthesis methods such as biosynthesis, targeted delivery systems, and combinations with antioxidants can potentially mitigate harmful effects [68,169,170,171].
- Therapeutic Monitoring: Therapeutic drug monitoring is less studied for AgNPs compared to traditional antimicrobials, making it harder to optimize AgNP dosage to minimize toxicity. The analysis of therapeutic drug monitoring of traditional antimicrobial agents helps to reduce their toxicity, although suboptimal concentrations may promote the development of resistance. In contrast, AgNPs demonstrate sustained antimicrobial activity at levels compatible with host cell viability [68]. The toxicity of AgNPs correlates with the particles’ size, agglomeration, and coating [172].
- Antimicrobial Activity: Antimicrobials are among the most prevalent agents responsible for drug-induced hepatic and renal injuries, many of which are dose-dependent, indicating that the toxicity is linked to the amount of administered drug. These agents frequently target specific bacterial pathways, although they also produce collateral damage to host cellular structures. For example, metronidazole causes the fragmentation of DNA and proteins. Some antimicrobial agents, such as aminoglycosides, are related to nephrotoxicity, while others, like fluoroquinolones, generate neurotoxicity, and some, like linezolid, even cause hematotoxicity [173]. On the contrary, AgNPs demonstrate sustained antimicrobial activity at levels compatible with host cell viability. For instance, serum-capped silver nanoparticles have shown high antimicrobial activity and a wide margin of safety for mammalian cells in a mice model [174].

Overall, both AgNPs and traditional antimicrobials have potential toxic effects. Hence, the cautious use of AgNPs is imperative, and adherence to prescribed safety protocols is crucial. This includes the use of personal protective equipment and the avoidance of prolonged exposure to high concentrations [148]. While the advantages of AgNPs are substantial, their potential toxicity and impact on human health must be thoroughly studied to establish appropriate regulations for their use [147].

## 7. Concluding Remarks

Silver nanoparticles (AgNPs) have demonstrated significant efficacy against a broad spectrum of infectious and non-infectious diseases. This review focuses particularly on their impact on bacteria and biofilms, highlighting mechanisms such as the disruption of cell membrane structure, the generation of ROS, and DNA damage.

Furthermore, this review underscores AgNPs’ potential in combating antibiotic resistance and infections associated with biofilms. Studies indicate that AgNPs effectively inhibit the growth of multidrug-resistant bacteria and facilitate the delivery of antibiotics within biofilms.

The use of AgNPs in non-infectious diseases is a burgeoning area of interest, with these materials showing versatile applications in conditions like diabetes, wound healing, and cancer. This review also discusses their anti-diabetic, anti-inflammatory, and antitumoral properties. AgNPs have shown promise in enhancing the therapeutic efficacy of chemotherapy and radiotherapy. Additionally, AgNPs serve as valuable tools not only in treatment, but also in disease detection, particularly as biosensors. The review also introduces the topic of AgNPs’ toxicity in humans.

However, the presented data still leave important questions unanswered that need to be addressed. These include investigating the precise mechanisms by which AgNPs induce ROS, modify proteins, and cause DNA damage in microorganisms; exploring the potential of AgNPs to target persistent cells; investigating new biomimetic targets and approaches to advance the clinical applications of AgNPs in tumor cells; or conducting a comprehensive characterization of AgNPs and developing new coatings to mitigate their potential toxicity. A more detailed understanding of the mechanisms of action of AgNPs will allow for the exploitation of their biomedical application potential.

## Figures and Tables

**Table 1:** Classification of size, morphology, and surface chemistry of AgNPs.

Category	Subcategory	Description
Size	Ultrasmall AgNPs	<10 nm, characterized by enhanced surface reactivity and potentially increased cellular uptake
	Small AgNPs	10–30 nm, generally exhibit significant antimicrobial effects, with efficacy often inversely proportional to size
	Medium AgNPs	30–60 nm, may display a balance between stability and biological activity
	Large AgNPs	60–100 nm, may exhibit reduced antimicrobial efficacy compared to smaller counterparts
Morphology	Nanospheres	Spherical morphology
	Nanocubes	Cubic morphology, often associated with enhanced antibacterial effects due to closer membrane interaction
	Nanowires	One-dimensional structures, exhibiting variable antimicrobial activity depending on aspect ratio and surface properties
	Nanorings	Ring-shaped morphology, demonstrating potentially superior activity in certain applications over prolonged periods
	Other Morphologies	E.g., nanoprisms, nanoplates, etc., exhibiting distinct properties and requiring further characterization of their biological activities
Surface modification/stabilization	Unmodified AgNPs	Lacking surface modifications or stabilizers
	Stabilizer-Coated AgNPs	Possessing a surface layer of stabilizing agents to prevent aggregation and influence electrokinetic characteristics
	Functionalized AgNPs	Modified with biologically active compounds to enhance cell membrane penetration or target specific biological entities

**Table 2:** Therapeutic properties, mechanisms of action, and references related to infectious pathologies.

Therapeutic Properties	Key Mechanisms of Action	References
Antibacterial properties	Disruption of cell wall and membrane integrity	[11,22]
	Inhibition of ATP synthesis and respiratory enzymes	[22,23,24,25]
	Denaturation of ribosomes	[22,23,24,25]
	Interference with bacterial signal transduction	[22,23,24,25]
	Generation of ROS	[22,23,24,25,26,27,28,29,30,31]
Antifungal properties	Increased intracellular ROS, leading to apoptosis	[28,32,33]
	Inhibition of hyphal growth and spore germination	[34]
	Membrane disruption via Ag^+^ release	[32,35]
	Inhibition of H^+^ ATPase and cellular respiration	[35]
	Alteration of tricarboxylic acid cycle and ergosterol synthesis	[36]
Antiprotozoa properties	Elevated ROS production, overwhelming parasite defenses	[37,38]
	Inhibition of cyst germination in Acanthamoeba	[39]
Antiviral properties	Prevention of viral penetration into host cells	[40,41]
	Binding to sulfur-rich domains of glycoproteins	[40,42,43]
	Damage to viral nucleic acids	[44,45]
	Competitive binding of AgNPs to cellular receptor sites	[41]
	ROS production and reduction in possibility of aggregation	[46]

**Table 3:** Therapeutic properties, mechanisms of action, and references related to non-infectious pathologies.

Therapeutic Properties	Key Mechanisms of Action	References
Anti-Diabetic	Insulin sensitization	[101]
	GLUT2 membrane translocation enhancement	[102]
	Pancreatic protection against ROS	[103,104]
	Advanced glycation end-product inhibition	[105,106,107,108,109]
Wound Healing	Prevent of microbial growth and improvement of oxygen delivery	[110]
	Modulation of immune response	[111]
	Re-epithelialization and differentiation of fibroblasts	[112]
Antitumoral properties	Mitochondrial apoptosis via ROS cascade	[113,114]
	Dysregulation of angiogenesis	[115,116]
	Changes in cell cycle	[13,117]
	Theranostic drug delivery enhancement	[118,119,120]

## Data Availability

Not applicable.
